# Sanfilippo syndrome registry project and natural history studies: an example of patients, parents and researchers collaborating for a cure

**DOI:** 10.1186/1750-1172-9-S1-P7

**Published:** 2014-11-11

**Authors:** Jill Wood, Stuart Siedman, Jennifer Siedman, Paul Levy, Kyle Brown, Kim McBride, Kevin Flanigan, Raquel Marques, Arleta Feldman, Robert PleHcha, Sean Ekins

**Affiliations:** 1 Jonah's Just Begun, P.O. Box 150057, Brooklyn, NY 11215, USA; 2 Ben's Dream, Sanfilippo Research Foundation, P.O. Box 81268, Wellesley, MA 02481---0002, USA; 3 The Children’s Hospital at Montefiore, MMC CHAM, 3415 Bainbridge Avenue, Bronx, NY 10467, USA; 4 Patient Crossroads, 4 West 4th Avenue, Penthouse C, San Mateo, CA 94402, USA; 5 Nationwide Children’s Hospital, 700 Children's Drive Columbus, Ohio 43205, USA; 6 Mały Maciek i Wielcy Czarodzieje OS. Mozarta 1/32, 31---232 Krakow, Poland; 7 RareConnect.org, A Partnership of Eurordis and NORD, Spain

## 

In the past 5 years two clinical trials for Sanfilippo type A have been conducted, namely Shire’s enzyme replacement therapy and Lysogene’s gene therapy. Nationwide Children’s Hospital is aiming to start gene-therapy clinical trials for types A & B in 2014. For other Sanfilippo types C and D the research is at preclinical stages including gene-therapy (Type C) and developing and characterizing a knock-out mouse model (Type D). These recent scientific advancements towards treatments for Sanfilippo Syndrome indicate that it is time for us to collect and analyze information on Sanfilippo patients in a single centralized registry as part of the PatientCrossroadsCONNECT website (https://connect.patientcrossroads.org/?org=SanfilippoRegistry) In addition it is important we understand how the disease progresses and what differences there may be between the different types of Sanfilippo Syndrome. This requires natural history studies (NHS) which can help us in determining the clinical outcome measures, identify potential surrogate endpoints via defined assessments including standardized clinical, biochemical, neurocognitive, behavioral, developmental, and imaging measures. From our experiences such data collected from NHS studies are generally not shared between researchers except when published as scientific papers at a much later date. Sanfilippo Syndrome has a very small patient population and the participation in multiple NHS (which may be occurring simultaneously) places an unrealistic burden on patients and families. Sanfilippo Syndrome is also ultra---rare and patients are geographically diverse. Providing patients and families with an outlet to find pertinent information pertaining to Sanfilippo, such as where Natural History Studies and clinical trials are taking place, or making themselves known by participating in a centralized registry, is essential. With the use of RareConnect platform (http://www.rareconnect.org/en/community/sanfilippo-syndrome) we hope to bring families from around the world closer together and give them access to information that they may not have access to otherwise.

We describe how the data collected from the NHS studies for Types A and B performed at Nationwide Children’s Hospital and for Type C at The Children’s Hospital at Montefiore will be available to other qualified institutions to prevent repetition. Such NHS studies and registries can also help in identifying participants for future clinical trials. We illustrate through this work how close collaborations between parent/patients led disease organizations and clinical researchers, is essential to ensure our limited funding and time is well spent as we try to identify treatments as quickly as possible.

